# Pharmacological Evaluation of 3-Carbomethoxy Fentanyl in Mice

**DOI:** 10.3390/ph4020233

**Published:** 2011-01-25

**Authors:** Sonja Vuckovic, Milica Prostran, Milovan Ivanovic, Ljiljana Dosen-Micovic, Katarina Savic Vujovic, Cedomir Vucetic, Marko Kadija, Zeljko Mikovic

**Affiliations:** 1 Department of Pharmacology, Clinical Pharmacology and Toxicology, School of Medicine, University of Belgrade, P.O. Box 38, 11129, Belgrade, Serbia; 2 Faculty of Chemistry, University of Belgrade, Belgrade, Serbia; 3 Institute for Orthopaedic Surgery and Traumatology, Clinical Center of Serbia, School of Medicine, University of Belgrade, Belgrade, Serbia; 4 Department of High-risk Pregnancies, University Clinic of Gynaecology and Obstetrics “Narodni front”, Belgrade, Serbia

**Keywords:** fentanyl, 3-carbomethoxy fentanyl, hot plate, rotarod, acute toxicity, mice

## Abstract

In many animal species, as well as in humans, high doses of fentanyl (F) produce marked neurotoxic effects, such as muscular rigidity and respiratory depression. The antinociception (hot-plate test), impairment of motor coordination (rotarod test) and acute toxicity of intraperitoneal newly synthesized analogs, (±)*cis*-3-carbomethoxy- fentanyl (C) and (±)*trans*-3-carbomethoxyfentanyl (T) were evaluated in mice. The compounds tested induced antinociception, impairment of performance on the rotarod, and lethality in a dose-dependent manner. The relative order of antinociceptive potency was similar to motor impairment potency, as well as lethality: F > C > T. Naloxone hydrochloride (1 mg/kg; sc) abolished all the effects observed, suggesting that they are mediated *via* opioid receptors, most probably of μ type. There were no significant differences between the therapeutic indices of F, C and T. It is concluded, the introduction of 3-carbomethoxy group in the piperidine ring of the fentanyl skeleton reduced the potency, but did not affect tolerability and safety of the compound.

## 1. Introduction

**Figure 1:**
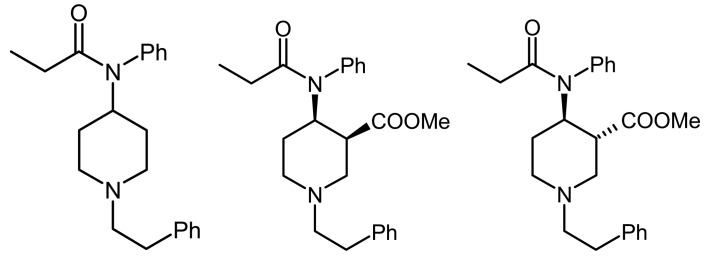
Fentanyl, (±)*cis*-3-carbomethoxyfentanyl and (±)*trans*-3-carbomethoxyfentanyl.

Fentanyl (Figure 1) is a strong and relatively short acting opioid drug widely used in surgery and in the treatment of postoperative and cancer pain [1]. However, in many animal species, as well as in humans, high doses of fentanyl and its analogs produce marked neurotoxic effects such as muscular rigidity and respiratory depression [1-4]. Since neurotoxic effects are clinically undesirable, the ability to synthesize the compound with less side effects could lead to the development of a new clinically valuable opioid drug [2,4-9].

In a previous study it has been found that introduction of 3-carbomethoxy group in the piperidine ring of fentanyl skeleton (Figure 1) provides less potent (∼2–10 x) opioid analgesics which possess rapid onset and a shorter duration of analgesia in rats [4,6-7]. In the present experiments, the neurotoxic effect such as impairment of motor coordination and lethality of intraperitoneal fentanyl (F) and newly synthesized fentanyl analogs, (±)*cis*-3-carbomethoxyfentanyl (C) and (±)*trans*-3-carbomethoxyfentanyl (T) were compared in mice.

## 2. Experimental

### 2.1. Animals

Male albino mice weighting 20–35 g obtained from Military Farm (Belgrade, Serbia) were used. All experiments were approved by the Institutional Animal Ethics Committee (Permission N 828/2/2-2, May 12^th^, 2009.) which operates in accordance with Revised Guide for the Care and Use of Laboratory Animals (NIH Guide, Volume 25, Number 28, 1996). The animals were housed in groups of 10 in plexiglas cages (36.5 × 21 × 14 cm) under standard conditions: temperature of 22 ± 1 °C, and a 12/12 h light/dark cycle with lights on at 08.00 h. Food pallets and tap water were available *ad libitum*, except during the experimental procedure. Prior to each experiment the animals were habituated to the handling and experimental procedures for at least three consecutive days. Experiments were done in a sound–proofed, diffusely illuminated room maintained at a temperature of 22 ± 1 °C. They were performed at the same time of the day between 9:00 and 13:00 h to avoid diurnal variation in behavioral tests. Experimental groups consisted of 6–10 mice. Each animal was used only once and was killed with i.p. injection of sodium thiopental.

### 2.2. Antinociception Testing

The antinociceptive activity was determined by the “hot plate” test (Technilab Instrument Inc. 475, Ugo Basile, Italy). The latency to a “hind paw lick or jump” response was measured for mice placed on a plate heated to 55 ± 0.5 °C [10]. In order to minimize tissue damage by repeated testing, a cut-off time of 30 s was adopted. This means that the maximum duration of a single exposure of rat to hot plate was 30 s. Predrug response latency was obtained 5 min before i.p. drug (or the saline in the control group) administration. In control and predrug latency testing, mice that did not respond to hot plate within 13 s were excluded from the experiment.

Postdrug response latency was measured after i.p. administration of test compound (or the saline in the control group) at 10, 20, 30, 40, 60, 80 and 100 min. The data are expressed quantally as the number of animals in which the antinociception was observed versus total number of animals receiving the same treatment. For antinociception, the following criterion was used: an antinociceptive effect was said to have occurred if postdrug response latency was doubled in respect to the control latencies. The ED_50_ value for analgesia (AD_50_) was defined as a dose at which 50% of testing animals doubled the control latencies.

### 2.3. Impairment of Motor Coordination Testing

Motor impairment was determined by rotarod test in mice according to Kinnard and Carr [11]. The test was undertaken by use of a rod of 3 cm diameter, rotating at a constant speed of 15 rpm (Treadmill for mice 7600, Ugo Basile, Milano, Italy). Two days before testing, mice were pretrained and only the animals able to remain on the rod for 60 s for two consecutive trials (separated by 30 min pause between trials) were used in the experiments. On the day of the experiment, the mice were tested once again to obtain the predrug time spent on the rotarod, which had to be at least 60 s for all animals. After the i.p. drug administration (or the saline in the control group), the number of animals per group unable to remain on the rod for at least 60 s was recorded at 5, 10, 15, 20, 40 and 60 min time points. The TD_50_ (the dose that induces motor impairment in 50% of animals tested) with 95% confidence limits was calculated from a corresponding quantal dose–response curve (Litchfield & Wilcoxon I procedure) according to Tallarida and Murray [12] by using the following criteria: an animal was said to be motorically impaired if it did not remain on the rotarod for the entire 60 s [11]. The ED_50_ value for impairment of motor coordination (TD_50_) was defined as a dose at which 50% of testing animals failed to remain on the rotarod for 60 s.

### 2.4. Acute Toxicity

Acute toxicity was examined with the different intraperitoneal doses of fentanyl, (±)*cis*-3 carbomethoxyfentanyl and (±)*trans*-3-carbomethoxyfentanyl in groups of 10 mice per dose. Lethal end points were closely monitored for the first 12 h, and after that, twice per day for 2 weeks. The LD_50_s (median effective doses for lethality) were calculated and compared according to the method of Litchfield and Wilcoxon [12].

### 2.5. Drugs Administration

Fentanyl citrate (ICN Yugoslavia, Belgrade, Serbia) and (±)*cis*- and (±)*trans*-3-carbomethoxy-fentanyl oxalate were dissolved in saline and injected i.p. at a final volume of 10 ml/kg. Both (±)*cis*-and (±)*trans*-3-carbomethoxyfentanyl (Faculty of Chemistry, University of Belgrade, Serbia) were examined as a racemic mixture. Doses of the drugs were calculated for the free base. Naloxone hydrochloride (Sigma Chemical Co. St. Louis, MO, USA) was also dissolved in saline, and injected sc (1 mg/kg) in the back before the i.p. injection of the test compound in the same volume. Saline alone (10 mL/kg) was administrated i.p. in a control group of rats.

### 2.6. Statistical Analysis

To permit direct comparison of different compounds and different effects, basic data for each animal were transformed to a quantal response (presence or absence of expected drug effect). For each effect and each dose maximum response obtained during the time of measurement was used for evaluation. Then, computations were done according to the methods of Tallarida and Murray [12]. First, the percentages of animals responding were converted to probits and plotted against the log dose. Then, the Litchfield & Wilcoxon procedure was used to calculate ED_50_ (median effective dose) values from corresponding quantal dose–response curves. Additionally, when data for a second quantal dose– response curve was entered, the same procedure calculated the potency ratio [with 95% confidence limits (95% CL)] for corresponding curves. In that way, relative potencies for fentanyl, (±)*cis*-3-carbomethoxyfentanyl and (±)*trans*-3-carbomethoxyfentanyl were calculated for antinociceptive, as well as toxic effects. Relative potency estimates were considered statistically significantly different when 95% CL did not overlap 1.0. Also, the potency ratios between two ED_50_s (TD_50_/AD_50_ and LD_50_/AD_50_) denoted as the TIs (therapeutic index), for each drug were determined. If the 95% confidence interval for a TI fails to include 1.0, then ED_50_s are significantly different.

The duration of action was determined as a time that is necessary for antinociception and motor impairment to cease in 50% of tested animals after i.p. injection of equi-effective doses (4 × ED_50_ for antinociception) of all three tested compounds. Percentages of responding rats for each effect and each compound was plotted as a function of time.

The drug response was compared to the control response at each time point of measurement by using Fisher's test. The difference in time-effect course between equi-antinociceptive doses of all three compounds tested was estimated by using both Fisher's and Mann-Whitney test. A P value of less than 0.05 was considered statistically significant.

## 3. Results and Discussion

**Figure 2:**
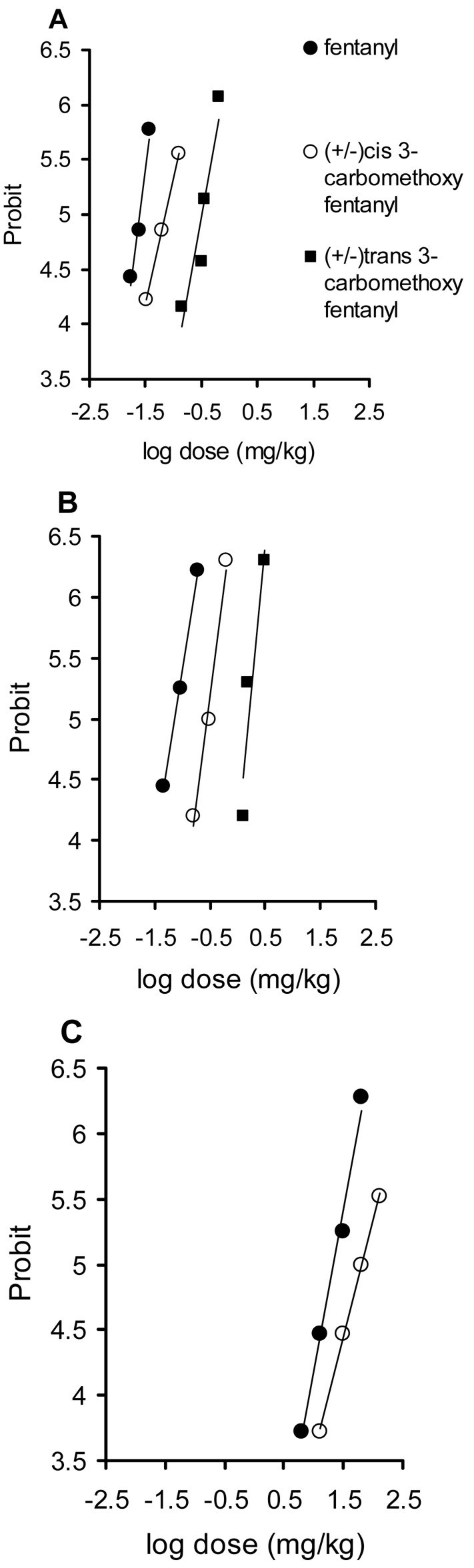
Log dose-probit curves for antinociception (hot plate) (A), motor impairment (rotarod test) (B) and lethality (C) for fentanyl, (±)*cis*-3-carbomethoxyfentanyl and (±)*trans*-3-carbomethoxyfentanyl, in mice, after i.p. drug administration. For antinociception, the following criterion was used: an antinociceptive effect was said to have occurred if postdrug response latency was doubled in respect to the control predrug latencies. Motor impairment was detected in the animal unable to maintain equilibrium on the rotating rod for 60 s. Each point represents the probit value obtained from at least 6 mice.

The present study showed that fentanyl and its newly synthesized analogs, (±)*cis*-3-carbomethoxy-fentanyl (C) and (±)*trans*-3-carbomethoxyfentanyl (T), caused a significant and dose-dependent antinociception, impairment of motor coordination and lethality in mice. For each effect tested, probit slopes for F, C and T are not significantly different (*p* > 0.05, test for parallelism) (Table 1; Figure 2). The relative potencies for F, C and T are presented in Table 1.

This is the first report on the toxicity of C and T. C- and T-induced antinociception in the hot plate test in mice is consistent with the ability of these drugs to reduce tail immersion latency in rats [4]. In comparison with F (0.017–0.037 mg/kg), the antinociceptive potencies of C (0.032–0.125 mg/kg), and T (0.145–0.631 mg/kg), were reduced by factor 3 and 14, respectively (*P* < 0.05) (Figure 2; Table 1). Also, in comparison with F (0.046–0.186 mg/kg), the potencies of C (0.155–0.619 mg/kg), and T (1.291–3.097 mg/kg) to induce the motor impairment, were reduced about 4 and 25 times, respectively (*P* < 0.05) (Figure 2; Table 1). Also, C (16.26–128.83 mg/kg) is about 3 times less potent in producing lethal effect than fentanyl (6.31–63.10 mg/kg) (*P* < 0.05) (Figure 2; Table 1). T is a significantly less potent drug than C (4.6 and 5.8 times for analgesia and motor impairment, respectively) (not shown). Each of the tested compounds, exhibited similar (*p* > 0.05) relative potencies in producing all effects tested (95% confidence intervals overlap) (Table 1). If a series of related agonists exhibits identical relative potencies in producing distinct effects, it is likely that these effects are mediated by similar or identical receptor molecules [13].

Both, antinociceptive and toxic effects, were abolished by pretreatment with naloxone hydrochloride (1 mg/kg), nonselective antagonist of opioid receptors (not shown), indicating that they are mediated by opioid receptors. In the case of F, C and T, they are of μ type, most probably. Most of the currently available strong opioids exert their analgesic and adverse effects primarily through the opioid μ receptors [14].

The therapeutic indices are shown in Table 2. As F, C, and T did not alter rotarod performance in the antinociceptive doses used, it is possible that the antinociceptive behavior was not due to motor impairment or sedation. All drugs tested demonstrated good separation between antinociceptive efficacy in a thermal pain model, and motor impairment induction. The TD_50_ values for F, C, and T are significantly greater than their AD_50_ values [2.9 (1.6–5.4), 4.0 (2.0–7.9) and 5.0 (3.0–8.5) times, respectively] (Table 2). The greater the TI value is, the greater is observed drug tolerability. There are no significant differences between therapeutic indices for F, C and T, which means that these compounds are equally tolerable in regard to motor impairment. These observations suggest that C and T are effective drugs in hot plate test in mice, in a dose range that is not related to motor impairment, and that in comparison with fentanyl C and T are less potent, but not less tolerable drugs.

**Figure 3:**
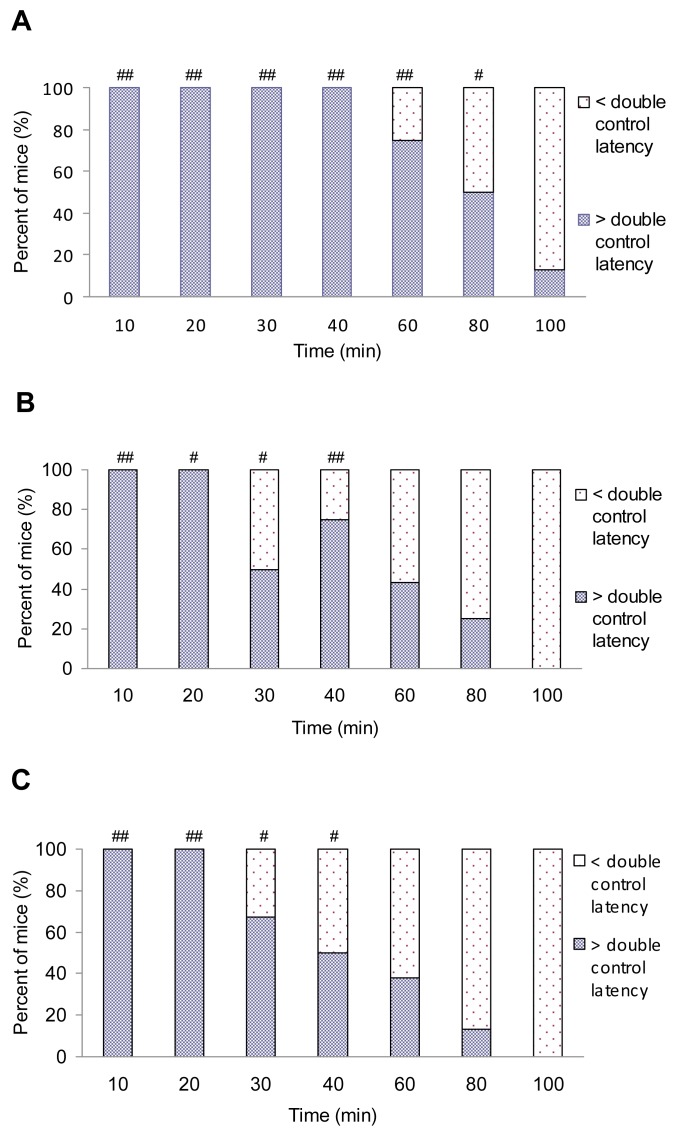
Time course of the antinociceptive effects of A) fentanyl, B) (±)*cis*-3-carbomethoxyfentanyl and C) (±)*trans*-3-carbomethoxyfentanyl in mice. The incidence of antinociception is plotted as a function of time after i.p. injection of equi-effective doses *i.e.* 4 × ED_50_ for antinociception of each drug. Each drug was tested by using 6-10 rats. Each point represents the percentage of rats that respond to the treatment with doubled postdrug response latency. Control animals (n = 7) received saline in the same volume. ^#^ *P* < 0.05 and ^##^ *P* <0.01 indicate a significant difference of the responses of fentanyl analogs in comparison to control (Fisher's test).

**Figure 4:**
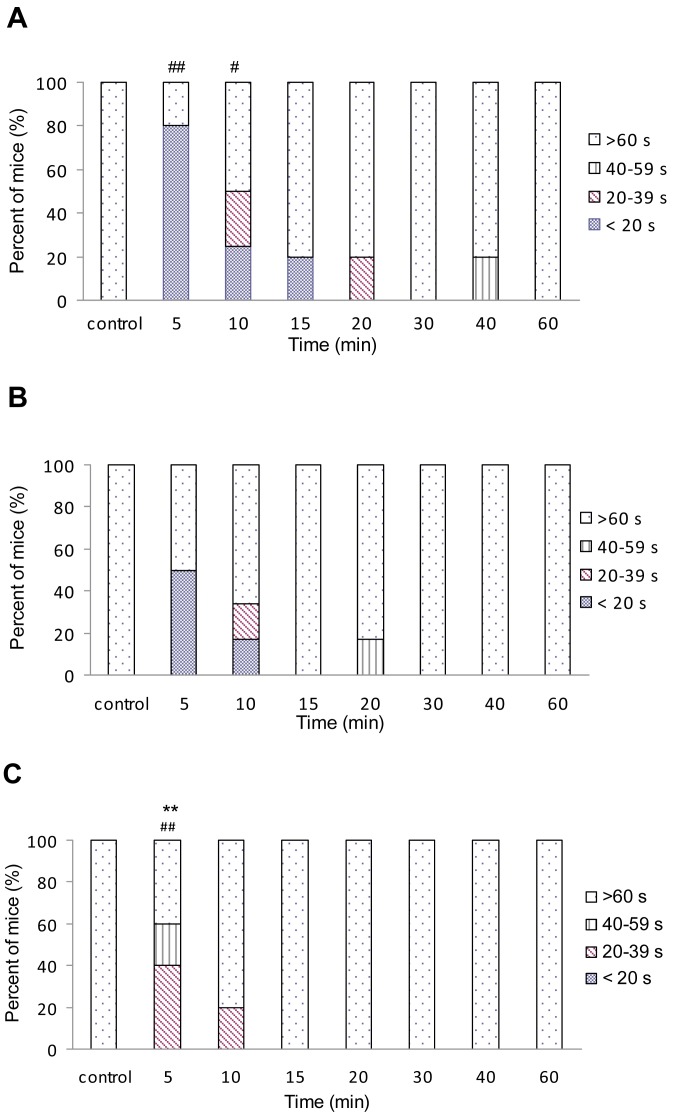
Time course of the effects of A) fentanyl, B) (±)*cis*-3-carbomethoxyfentanyl and C) (±)*trans*-3-carbomethoxyfentanyl on motor performance of mice. The incidence of motor impairment is plotted as a function of time after i.p. injection of equi-effective doses *i.e.* 4×ED_50_ for antinociception of each drug. Each drug was tested by using 6–10 rats. Each point represents the percentage of rats that respond according to criterion: >20, 20–39, 40–59 and >60 s spent on rotarod. * *P* < 0.05 and ** *P* < 0.01 (Mann-Whitney test) indicate a significant difference of the responses of (±)*cis*- or *trans*-3-carbomethoxy-fentanyl in comparison to fentanyl. ^#^ *P* < 0.05 and ^##^ *P* < 0.01 indicate a significant difference of the responses of fentanyl analogs in comparison to control (Fisher's test).

By using equi-antinociceptive doses (4 × ED_50_), C and T produced shorter duration of action than F. The duration of actions (time that is necessary for drug response to cease in 50% of tested animals) were approximately 80, 40 and 40 min (antinociception) and 10, 5 and 5 min (impairment of motor coordination) for F, C and T, respectively. The duration of antinociception of C and T was not statistically different from F (*p* > 0.05; Mann-Whitney test), but the duration of motor impairment of T was statistically reduced (*P* < 0.01) in comparison with F (Figure 3 and 4). This is not the case with C. In our previous experiments it has been clearly shown that C and T given i.p. in rats exhibited significantly shorter antinociception in comparison with F [4]. On that occasion, the differences in duration of action were explained by a different pharmacokinetics between fentanyl and 3-carbomethoxy fentanyl. Moreover, the present finding suggests species differences in the pharmacokinetics of 3-carbomethoxyfentanyl.

Acute toxicities of F and C have been evaluated in mice. The animals have been followed up for 14 days. There is no difference between LD_50_ values obtained after 24 h and 14 days. Signs and symptoms of acute toxicity of all three drugs were similar: dyspnea, hypoventilation, cyanosis, convulsion, Straub tail, trunk muscle rigidity, prostration *etc.* In agreement with opioid activity, respiratory depression was the most prominent symptom and probably the main cause of death, as well [15]. This is supported by fact that most death occurred in the first few hours after drug administration. Necropsy has not been performed. There is no significant difference between the therapeutic indices [median lethal dose (LD_50_) / median antinociceptive dose (AD_50_)] between F and C: 858.1 (444.9–1654.7) and 918.5 (392.8–2147.5), respectively (Table 2). This finding suggested that F and C are equally safe in mice.

## 4. Conclusion

In conclusion, the antinociceptive and toxic effects of newly synthesized fentanyl analogs, (±)*cis*-3-carbomethoxyfentanyl and (±)*trans*-3-carbomethoxyfentanyl are mediated *via* opioid receptors, most probably of μ type. The introduction of 3-carbomethoxy group in the piperidine ring of the fentanyl skeleton reduced the potency, but did not affect tolerability and safety of the compound.

## Figures and Tables

**Table 1:** Median effective doses for antinociception (AD_50_), impairement of motor coordination (TD_50_) and lethality (LD_50_), correlation coefficient (r), probit slopes and relative potencies with 95% confidence limits (95% CL) for fentanyl, (±)*cis*-3-carbomethoxyfentanyl and (±)*trans*-3-carbomethoxyfentanyl in inducing antinociception, impairement of motor coordination and lethality in mice.

**Effect**	**AD_50_ or TD_50_ or LD_50_ (95% confidence interval)**	**r**	**Probit slope**	**Relative potency**
**Fentanyl** (0.017–63.096 mg/kg)				

**Antinociception ^1^**	0.025 (0.018–0.034)	0.979	3.9 (−6.4–14.3)	1
**Impairement of motor coordination ^2^**	0.073 (0.043–0.123)	0.999	2.9 (1.3–4.5)	1
**Lethality**				
**24 h**	26.30 (14.78–46.77)	0.990	2.0 (1.1–2.9)	1
**14 days**	21.25 (11.99–37.67)	0.994	2.5 (1.6–3.3)	1

**(±)*cis*-3-Carbomethoxyfentanyl** (0.032–128.825 mg/kg)				

**Antinociception ^1^**	0.071 (0.040-0.124)	1	2.3 (1.7–2.8)	0.35* (0.18–0.67)
**Impairement of motor coordination ^2^**	0.282 (0.188-0.421)	0.993	3.5 (−1.8–8.9)	0.26* (0.13–0.50)
**Lethality**				
**24 h**	82.71 (43.73–156.46)	0.981	1.8 (0.7–2.9)	0.32 * (0.13–0.75)
**14 days**	65.12 (34.51–122.85)	0.999	1.8 (1.6–2.0)	0.33 * (0.14–0.77)

**(±)*trans*-3-Carbomethoxyfentanyl** (0.145–3.097 mg/kg)				

**Antinociception ^1^**	0.32 (0.21–0.50)	0.994	2.9 (−1.2–7.1)	0.08* (0.01–0.58)
**Impairement of motor coordination ^2^**	1.63 (1.20–2.20)	0.949	4.9 (−15.6–25.3)	0.04* (0.01–0.24)

1 criterion > double control latency;

2 criterion < 60 s, AD_50_s, TD_50_s and LD_50_ were calculated by using at least 3 doses of testing compound. One dose is tested in 6-10 mice (Litchfield &Wilcoxon test) [12];

* Relative potency estimates were considered statistically significant when 95% CL did not overlap 1.0 (*P* < 0.05, Litchfield & Wilcoxon II test) [12].

**Table 2:** Therapeutic indices for fentanyl, (±)*cis* 3-carbomethoxy fentanyl and (±)*trans* 3-carbomethoxy fentanyl in mice.

**Compound**	**Fentanyl**	**(±)*cis*-3-carbomethoxy-fentanyl**	**(±)*trans*-3-carbomethoxy-fentanyl**
**Toxicity**	**Therapeutic index** (TI = TD_50_/AD_50_ or LD_50_/AD_50_)	**Therapeutic index** (TI = TD_50_/AD_50_ or LD_50_/AD_50_)	**Therapeutic index** (TI = TD_50_/AD_50_ or LD_50_/AD_50_)
**Impairment of motor**	2.9	4.0	5.0
**coordination (rotarod)^2^**	(1.6–5.4)	(2.0–7.9)	(3.0–8.5)
**Lethality**	1061.8	1166.7	/
**(24 h)**	(548.9–2053.8)	(497.9–2733.2)	
**(14 days)**	858.1	918.5	
	(444.9–1654.7)	(392.8–2147.5)	

1 criterion > double control latency;

2 criterion < 60 s; AD_50_ = median effective dose for antinociception; TD_50_ = median effective dose for impairment of motor coordination (median toxic dose); LD_50_ = median lethal dose; Potency ratios TD_50_/AD_50_ and LD_50_/AD_50_ denoted as the TIs (therapeutic indices), for each drug were determined. If the 95% confidence interval for a TI fails to include 1.0, then ED_50_s are significantly different.
